# A Unique Presentation of Acute Immune Thrombocytopenia Secondary to Helicobacter pylori Infection

**DOI:** 10.7759/cureus.52220

**Published:** 2024-01-13

**Authors:** Kyrillos Girgis, Allen George, Leandro Gutierrez, Jacob Brown, Rafail Beshai

**Affiliations:** 1 Internal Medicine, Newark Beth Israel Medical Center, Newark, USA; 2 Cardiovascular Disease, Virtua Health, Camden, USA; 3 Internal Medicine, Jefferson Health, Stratford, USA

**Keywords:** low platelets, dyspepsia, rare cause, immune thrombocytopenia, helicobacter pylori

## Abstract

Thrombocytopenia, a condition characterized by low platelet counts, can arise from various causes, including autoimmune diseases. Immune thrombocytopenia (ITP), a diagnosis made by excluding other possible causes, is categorized as primary or secondary, with primary ITP being idiopathic and secondary ITP associated with infections or autoimmune conditions. This study highlights a unique instance of severe thrombocytopenia triggered by *Helicobacter pylori* infection.

## Introduction

Immune thrombocytopenia (ITP) is an autoimmune disorder characterized by the destruction of normal platelets by the immune system, leading to insufficient platelet production and bleeding [1]. ITP can be primary or secondary, with primary ITP having no identifiable cause and secondary ITP being linked to autoimmune conditions, medications, genetics, or, most commonly, infections. Common infections associated with ITP include hepatitis B and C, and human immunodeficiency virus (HIV) [2]. While previous studies have primarily focused on chronic ITP associated with *Helicobacter pylori*, this study presents a unique instance of a young female developing ITP shortly after an *H. pylori *diagnosis.

## Case presentation

A 20-year-old female with a history of dyspepsia for one month was referred to our emergency room due to "low blood levels" by her primary care physician. The patient reported symptoms of vomiting bright red blood, easy bruising on her upper extremities, prolonged epistaxis, bleeding gums during tooth brushing, and dark stools, all occurring within one week of her *H. pylori *diagnosis. She had initiated triple therapy, consisting of amoxicillin 500 mg twice daily, clarithromycin 500 mg twice daily, and pantoprazole 40 mg twice daily, for *H. pylori *treatment.

Upon presentation, her vital signs showed a heart rate of 78 bpm, temperature of 98.5°F, respiratory rate of 18 breaths per minute, oxygen saturation of 99% on room air with elevated blood pressure of 136/101 mmHg. Physical examination revealed bilateral 1-2 mm petechiae on her upper and lower extremities and scattered purpuras on her torso. No gingival bleeding was observed.

Initial laboratory results showed severe thrombocytopenia with a platelet count of 3,000 × 10^9^/L with normal hemoglobin and white cell counts (Table 1). Basic metabolic panel shows normal electrolytes except for hypokalemia of 3 mmol/L and normal renal functions.

**Table 1:** Lab results showing severe thrombocytopenia.

Lab test	Lab value	Reference range
Complete blood count		
White blood cells	8.5 × 10^9^/L	4-10 × 10^9^/L
Hemoglobin	14 g/dL	12.5-16.5 g/dL
Platelets	3 × 10^9^/L	150-400 × 10^9^/L
Coagulation panel		
Prothrombin time	13.5 s	9.4-12.5 s
Partial thromboplastin time	31.9 s	25.1-36.5 s
Basic metabolic panel		
Sodium	139 mmol/L	135-145 mmol/L
Potassium	3 mmol/L	3.5-5 mmol/L
Chloride	107 mmol/L	95-105 mmol/L
Calcium	9.2 g/dL	8.5-10.1 g/dL
Bicarbonate	25 mmol/L	21-32 mmol/L
Glucose	90 mg/dL	75-105 mg/dL
Blood urea nitrogen	7 mg/dL	7-18 mg/dL
Creatinine	0.6 mg/dL	0.7-1.3 mg/dL
Thyroid-stimulating hormone	0.5 IU/mL	0.35-3.5 IU/mL

Due to the risk of spontaneous bleeding, the patient was admitted to the ICU for close monitoring. During her ICU stay, a comprehensive workup ruled out other causes of thrombocytopenia, including negative screenings for systemic infections, such as HIV 1/HIV 2 and hepatitis B and C. Rheumatological workup including anti-nuclear antibody, anti-double-stranded DNA antibody, rheumatoid factor and anti-cyclic citrullinated peptide antibody, anti-scleroderma 70 antibody, anti-Sjögren's-syndrome-related antigen A (anti-SSA) and anti-Sjögren's-syndrome-related antigen B (anti-SSB) autoantibodies was negative.

The patient received a diagnosis of secondary ITP induced by *H. pylori*. Treatment consisted of intravenous dexamethasone 40 mg daily for four days and intravenous immunoglobulin (IVIG) 75 mg daily for two days, targeting dosing of 1 mg/kg for ITP. The patient's platelet count improved from 3,000 to 17,000 × 10^9^/L the following day and continued to increase. After completing the prescribed course of steroids and immunoglobulin, her platelet count reached 400,000 × 10^9^/L.

Subsequently, the patient was transferred from the ICU to the general medical floor, where her platelet count remained stable. She resumed her *H. pylori *treatment regimen, with her platelet count remaining consistently 400,000 × 10^9^/L, reaffirming the diagnosis of secondary immune thrombocytopenia linked to *H. pylori* infection.

## Discussion

Immune thrombocytopenic purpura (ITP) is characterized by isolated thrombocytopenia (platelet count <100,000/μL), a normal white blood cell count, and normal hemoglobin, often accompanied by a generalized purpuric rash [3]. ITP is classified as primary (without an underlying cause) or secondary (associated with conditions like systemic lupus erythematosus {SLE}, HIV, or drug-induced) [3]. Data on the prevalence of *H. pylori *infection in adult ITP patients do not differ significantly from those of the general population matched for age and geographical area. Investigations using a 13C-urea breath test as a detection method have been conducted, primarily in regions like Japan and Italy, where *H. pylori *prevalence rates are high [4].

The exact mechanism behind *H. pylori*-associated thrombocytopenia remains unclear, but several theories exist. *H. pylori *may activate Fcγ receptors on macrophages and monophages. Additionally, *H. pylori *components may mimic platelet antigens, leading to increased platelet uptake and phagocytosis [5]. This aligns with the process of immune thrombocytopenia, which involves antiplatelet antibodies accelerating platelet destruction and reducing production [5]. Other infectious agents, such as the human immunodeficiency virus and hepatitis C virus, have been associated with ITP, further supporting the role of infectious agents in triggering autoimmune responses leading to ITP [3]. In 1988, a study in Japan reported improvements in platelet counts in individuals treated for *H. pylori *compared to those who did not receive treatment, with a resolution of ITP in 63% of cases after *H. pylori *eradication [2].

In the present case, the possibility of amoxicillin or proton pump inhibitor-induced ITP is low, as the platelet count remained consistently in the 400,000s/µL with the resumption of the triple therapy and discontinuation of the steroids and the IVIG. A prospective study was done to prove the importance of *H. pylori* eradication therapy in the treatment of *H. pylori*-induced ITP. In that study, 37 ITP patients were treated with triple therapy regardless of *H. pylori *infection. At 24 weeks, 16 of 26 *H. pylori*-positive patients (62%) had a therapeutic response defined as a platelet count >100,000/µL, while none of the *H. pylori*-negative patients had a response [4].

## Conclusions

*H. pylori* has been considered a potential cause of chronic immune thrombocytopenia. The present case illustrates a unique presentation of acute immune thrombocytopenia related to *H. pylori*. Clinicians should consider *H. pylori *as a potential cause in ITP cases lacking an identifiable etiology, emphasizing the importance of eradication therapy for ITP induced by *H. pylori*. Further studies are needed to elucidate the precise pathophysiology of *H. pylori*-induced ITP.
